# Integrative Temporo-Spatial, Mineralogic, Spectroscopic, and Proteomic Analysis of Postnatal Enamel Development in Teeth with Limited Growth

**DOI:** 10.3389/fphys.2017.00793

**Published:** 2017-10-24

**Authors:** Mirali Pandya, Hui Liu, Smit J. Dangaria, Weiying Zhu, Leo L. Li, Shuang Pan, Moufida Abufarwa, Roderick G. Davis, Stephen Guggenheim, Timothy Keiderling, Xianghong Luan, Thomas G. H. Diekwisch

**Affiliations:** ^1^Texas A&M Center for Craniofacial Research and Diagnosis, Dallas, TX, United States; ^2^Brodie Laboratory for Craniofacial Genetics, University of Illinois at Chicago, Chicago, IL, United States; ^3^Department of Chemistry, University of Illinois at Chicago, Chicago, IL, United States; ^4^Medicine, University of Michigan, Ann Arbor, MI, United States; ^5^Proteomics Center of Excellence, Northwestern University, Evanston, IL, United States; ^6^Department of Earth and Environmental Sciences, University of Illinois at Chicago, Chicago, IL, United States

**Keywords:** enamel, hydroxyapatite, X-ray powder diffraction, proteomics, amelogenin

## Abstract

Tooth amelogenesis is a complex process beginning with enamel organ cell differentiation and enamel matrix secretion, transitioning through changes in ameloblast polarity, cytoskeletal, and matrix organization, that affects crucial biomineralization events such as mineral nucleation, enamel crystal growth, and enamel prism organization. Here we have harvested the enamel organ including the pliable enamel matrix of postnatal first mandibular mouse molars during the first 8 days of tooth enamel development to conduct a step-wise cross-sectional analysis of the changes in the mineral and protein phase. Mineral phase diffraction pattern analysis using single-crystal, powder sample X-ray diffraction analysis indicated conversion of calcium phosphate precursors to partially fluoride substituted hydroxyapatite from postnatal day 4 (4 dpn) onwards. Attenuated total reflectance spectra (ATR) revealed a substantial elevation in phosphate and carbonate incorporation as well as structural reconfiguration between postnatal days 6 and 8. Nanoscale liquid chromatography coupled with tandem mass spectrometry (nanoLC-MS/MS) demonstrated highest protein counts for ECM/cell surface proteins, stress/heat shock proteins, and alkaline phosphatase on postnatal day 2, high counts for ameloblast cytoskeletal proteins such as tubulin β5, tropomyosin, β-actin, and vimentin on postnatal day 4, and elevated levels of cofilin-1, calmodulin, and peptidyl-prolyl cis-trans isomerase on day 6. Western blot analysis of hydrophobic enamel proteins illustrated continuously increasing amelogenin levels from 1 dpn until 8 dpn, while enamelin peaked on days 1 and 2 dpn, and ameloblastin on days 1–5 dpn. In summary, these data document the substantial changes in the enamel matrix protein and mineral phase that take place during postnatal mouse molar amelogenesis from a systems biological perspective, including (i) relatively high levels of matrix protein expression during the early secretory stage on postnatal day 2, (ii) conversion of calcium phosphates to apatite, peak protein folding and stress protein counts, and increased cytoskeletal protein levels such as actin and tubulin on day 4, as well as (iii) secondary structure changes, isomerase activity, highest amelogenin levels, and peak phosphate/carbonate incorporation between postnatal days 6 and 8. Together, this study provides a baseline for a comprehensive understanding of the mineralogic and proteomic events that contribute to the complexity of mammalian tooth enamel development.

## Introduction

Enamel development is an integral process of symphonic dimensions that is characterized by a continuous interplay between cells, matrices, minerals, proteins, and signals over the entire period of amelogenesis. Allegorically speaking, the key players in this symphony have been known for decades, including a mineral section that undergoes a transition from amorphous calcium phosphate and a protein section made up by classic enamel proteins such as amelogenins, ameloblastin, and enamelin, as they are further processed by enamel-related enzymes, including MMP20 and KLK4. As amelogenesis progresses, the volume percentage of proteins and water decreases, while the mineral content increases, resulting in a 96% mineral content in the mature enamel layer of adult mammals (Deakins, 1942; Stack, 1960; Robinson et al., 1978, 1979, 1988). Changes in the metastable enamel matrix that result from the loss of water and proteins have even been described as a “kind of crisis” (Eastoe, 1979), referring to the multiple effects of water and protein resorption on the interface between remaining proteins and maturing enamel crystals.

For decades, the effect of individual enamel proteins such as amelogenin on enamel crystal growth have been a most intriguing and rewarding subject of study (Lagerström et al., 1991; Diekwisch et al., 1993; Gibson et al., 2001; Iijima and Moradian–Oldak, 2004; Jin et al., 2009; Gopinathan et al., 2014). In addition to deciphering individual aspects of amelogenin function, much progress has been made elucidating the role of the less prominent enamel-related proteins ameloblastin and enamelin on enamel crystal growth and habit (Masuya et al., 2005; Lu et al., 2011; Hu et al., 2014). Moreover, it has been demonstrated that enamel proteins undergo posttranslational processing by the enamel proteinases MMP20 and KLK4 (Bartlett and Simmer, 1999; Simmer and Hu, 2002; Bartlett, 2013).

While much is known about the major proteins and minerals involved in tooth enamel formation, it has become increasingly obvious that amelogenesis is more complex than a mixture of an aqueous enamel protein solution with a combination of calcium and phosphate ions, subjected to enzymatic protein digestion and gradual removal of water over time. Recent studies have illustrated the importance of ion transport mechanisms for mineral transport (Hubbard, 2000; Paine et al., 2001; Lacruz, 2017), the effect of pH modulation through regulatory molecules (Takagi et al., 1998; Lacruz et al., 2010; Moradian-Oldak, 2012; Robinson, 2014) and the role of junctional proteins such as cadherins for ameloblast movement (Bartlett and Smith, 2013; Guan et al., 2014). These molecules and events are only one part of a process that ensures a gradual deposition of minerals at the dentin-enamel junction throughout amelogenesis and their step-wise conversion into hydroxyapatite crystals and alignment into enamel prisms into one of the most fascinating biomaterials found in nature.

Development of a synthetic or mimetic model of amelogenesis would greatly benefit from a temporo-spatial integration of the multitude of processes involved in mammalian amelogenesis. Such multi-level and multi-scale data mining commonly requires a systems biology approach. Systems biology of development seeks to integrate bioinformatic data analysis with other molecular, cellular, and tissue-related information to reach a higher-level, multifaceted, and integrative understanding of developmental processes (Bard, 2007; Edelman et al., 2009). Integrative approaches toward biological problems have become possible as a result of recent advances in bioinformatics and omics technologies, including proteomics, transcriptomics, and metabolomics (Mochida and Shinozaki, 2011). In mineralized tissue biology, systems biology would need to integrate genetic and proteomic data with mineralogic, structural, and spectroscopic data to develop a multi-dimensional understanding of a complex process such as amelogenesis.

In the present study we have employed first mandibular mouse molar amelogenesis as a model system to systematically map proteomic, spectroscopic, temporo-spatial, and mineralogic events during the first 8 days of postnatal enamel development. The benefit of a model based on teeth with limited growth is the synchronicity of developmental events leading up to maturation of the entire tooth surface by the time of tooth eruption and providing a homogeneous enamel matrix at each stage ideally suited for proteomic and spectroscopic analysis. During the course of this study we have generated sets of spectroscopic, proteomic, and mineralogic data and integrated related events through their common timescale of development. Our analysis provides timing of events, novel proteomic and spectroscopic data, and identification of novel non-hydrophobic groups of proteins and individual proteins that may contribute toward amelogenesis. Future studies will enhance our understanding of the interconnectedness between these processes during the progression of amelogenesis as they contribute to the formation of highly organized tooth enamel.

## Materials and methods

### Vertebrate animals and tissue preparation

**Figure 1:**
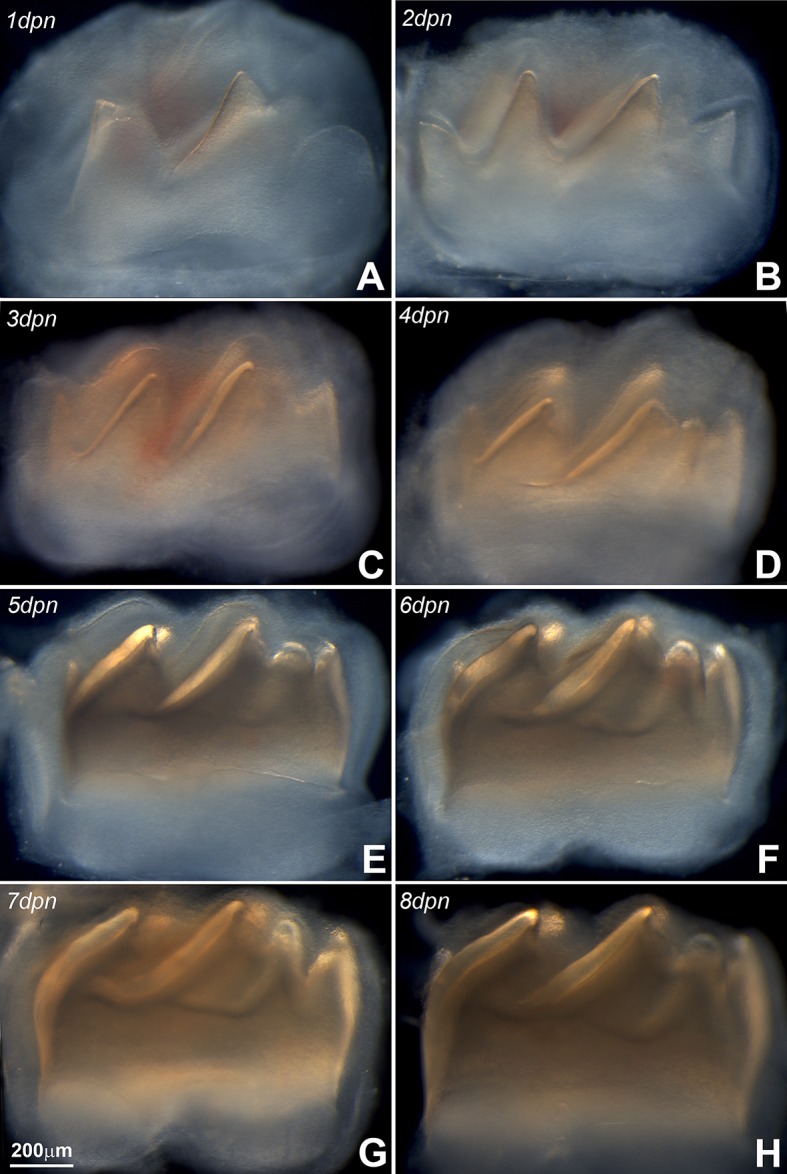
Panel **(A)** represents the mandibular molar at postnatal day (pnd) 1, **(B)** is pnd 2, **(C)** is pnd 3, **(D)** is pnd 4, **(E)** is pnd 5, **(F)** is pnd 6, **(G)** is pnd 7, and **(H)** is pnd 8. During the first 8 days of postnatal mouse molar development, the mineralized portion of the crown dentin continuously increased in height, while the length of the crown remained fairly unchanged. Note the gradual increase in the thickness of the enamel layer (identified in Figure 2F).

**Figure 2:**
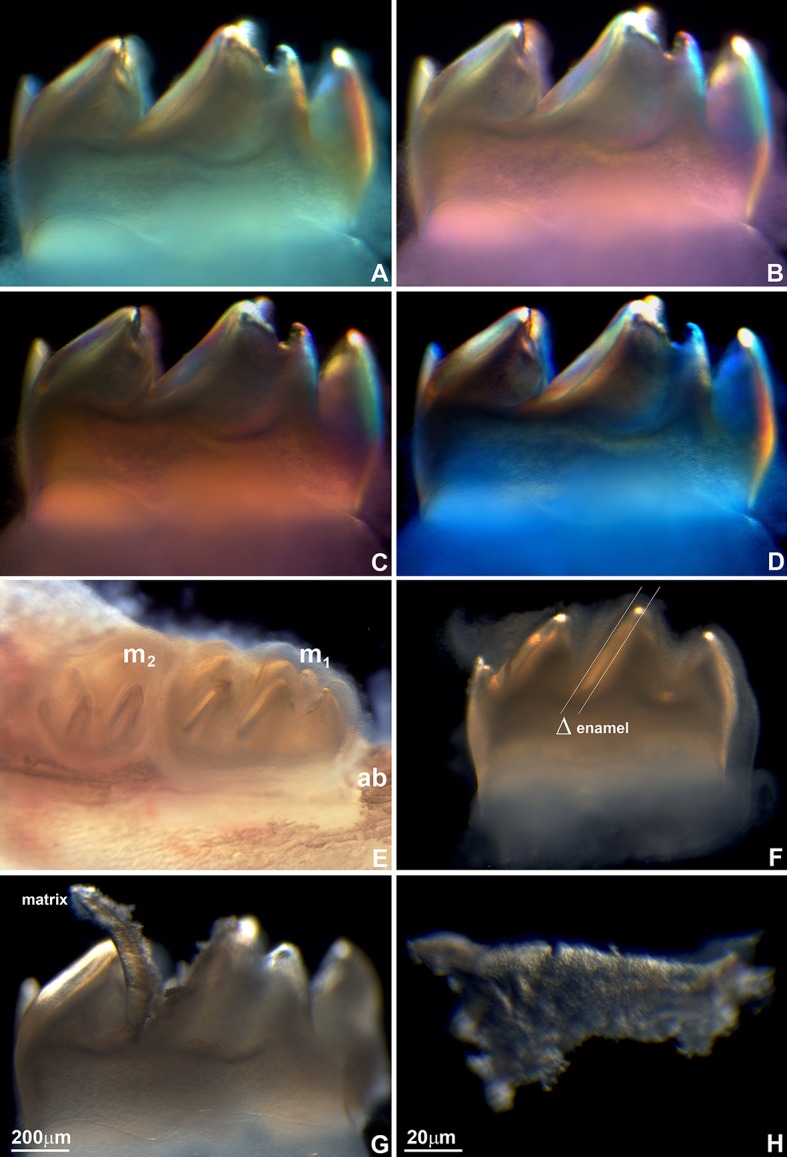
Mouse molar enamel birefringence **(A–D)** and enamel matrix layer dissection for mineral and protein analysis **(E–H). (A–D)** Mouse molars were dissected from mouse jaws and placed between crossed polarizers. Polarizers were oriented at the following angles: 0 degrees **(A)**, 90° **(B)**, 180° **(C)**, and 270° **(D)**. **(E)** Position of the first (m_1_) and the second (m_2_) mouse mandibular molar relative to the jaw bone and the alveolar bone (ab). **(F)** Enamel layer on the distal slope of the middle cusp of the first mandibular mouse molar used for the present analysis. Δ marks the thickness of the enamel layer between two parallel lines. **(G)** Preparation of the matrix layer from the distal slope of the middle cusp of the first mandibular mouse molar. **(H)** High magnification light micrograph of the dissected enamel layer.

First mandibular molars of 1, 2, 3, 4, 5, 6, 7, and 8 day postnatal mice (Figures 1A–H) were dissected from alveolar bone crypts (Figure 2E) to characterize the developing enamel matrix. Postnatal days 1–8 were used for Western blot. Days 2, 4, and 6 were selected for proteomics analysis. Days 1, 2, 4, 6, and 8 were used for X-ray powder diffraction, tissue dissection, and polarized microscopy. All animal experiments were approved by the IACUC committees at the University of Illinois, Chicago and Texas A&M College of Dentistry.

### Enamel thickness measurements and polarized light microscopy

Enamel thickness was measured on enlarged micrographs generated by a Leica stereo microscope. Enamel thickness was determined by calibration against a metric scale bar imaged at the same magnification. Birefringence of the enamel matrix was assessed by placing the enamel organs between crossed polarizers. The first polarizer was placed between the light source and the tooth organ and the second polarizer was placed between the tooth organ and the camera. The second polarizer was rotated in 45° intervals, and birefringence was identified based on the color changes within the enamel matrix.

### X-ray powder diffraction

For this study, the tooth enamel matrices of molars from days 1 to 8 postnatal mice were analyzed using X-ray powder diffraction in 1-day intervals (excluding samples from 3 day postnatal mice). For each day of analysis, the enamel matrix of four different mouse molars was harvested in distilled water, and samples from all four teeth at each developmental stage were pooled for further analysis. Samples were stored for about 24 h at < 0°C until analyzed at room temperature. Debye-Scherrer data simulations were obtained using a Bruker three-circle (transmission-mode) diffractometer using Mo radiation (0.7107 Å, APEX CCD detector, graphite monochromator, 0.3 mm Monocap capillary collimator, at operating conditions of 45 kV, 25 mA). Analyzed samples were~0.1 mm^3^ in volume, with each sample mounted on the end of a glass fiber, placed in the X-ray beam and rotated 360° about the glass-fiber axis. Detector positions were at two theta = 0, 20, and 35° using a frame resolution of 1,024 × 1,024 pixels, sample-to-detector distance of 120 mm, and each exposure was for 1,200 s. Data collection using the SMART collection software (Braintree, MA) and initial data processing using the Bruker GADDS software package (Bruker, Billerica, MA). Integration along the Debye rings was performed after data collection with a step (bin) size of 0.02, followed by construction of intensity vs. two-theta plots for each of the three detector positions, followed by a merger of the three plots based on the overlap of adjacent exposures to produce a traditional powder diffraction pattern. Additional pattern processing and phase identification using the Internal Center for Diffraction Data (ICDD, Newtown Square, PA, 2010) powder diffraction file was applied using the JADE software (Materials Data, Inc., Livermore, CA, 2009). Details of the Debye-Scherrer technique have been published in Klug and Alexander (1954) and the use of the Bruker three-circle diffractometer to simulate the Debye-Scherrer technique have been previously reported (Guggenheim, 2005).

### Attenuated total reflectance fourier transform infrared (ATR-FTIR) spectra

For FTIR analysis, mice molar enamel matrix samples were transferred to an ATR crystal (PIKE MIRacle single reflection diamond ATR) accessory placed in an FTIR (Bruker Vertex 80) sample compartment. Samples were pressed against the crystal surface with a pressure clamp to form a better contact covering most of the crystal surface. Sample absorbance spectra over the range 4,500–600 cm^−1^ were collected as an average of 2,048 scans (10 kHz scan speed with a DTGS detector) and processed with 3-term Blackman-Harris apodization and zero filling of 2. Background spectra, collected with same measurement parameters but without sample on the ATR crystal surface, were subtracted as a baseline correction.

### Proteomics sample preparation

Distal slope enamel matrices of 50 first mouse mandibular molars were prepared from 2, 4, and 6 dpn mice, and four sets of samples per time point were chosen. Enamel matrices from mice earlier than 2 dpn were not harvested due to a lack of overall quantity and enamel matrices from mice later than 6 dpn were omitted due to the advanced mineralization of those samples. Following cold acetone/trichloric acid precipitation, samples were redissolved in fifty microliters of 8 M urea. Reduction and alkylation of cysteines was accomplished by adding 1/10 volume of 45 mM DTT to the sample, followed by 45 min incubation at 37°C. After samples were cooled to room temperature, 1/10 volume of 100 mM iodoacetamide were added to the solution and samples were placed in dark at room temperature for 30 min. To equilibrate the sample for trypsin digestion, sufficient water was added to dilute the original 8 M urea/0.4 M ammonium bicarbonate solution 4-fold. A total of 1 μg trypsin was added and the sample was incubated at 37°C for 18–24 h. The trypsin digest was stopped by freezing until nanoLC-MS/MS analysis.

### Nano-scale liquid chromatography

Nano-scale liquid chromatography was performed using a Dionex Ultimate 3000 system. Mobile phase A was water/acetonitrile (95:5) with 0.1% formic acid. Mobile phase B was water: acetonitrile (5:95) with 0.1% formic acid. Digested sample was loaded offline onto a Thermo Scientific C18 PepMap100 peptide trap (300 μm ID × 5 mm, 5 μm, 100 A) with 100% mobile phase A flowing at 50 μL per minute. After allowing the peptides to concentrate and desalt for 10 min., the trap was switched inline with an Agilent Zorbax 300SB C18 nanoLC column (3.5 μm, 150 mm × 75 μm ID). The peptides were then resolved using a linear gradient from 5% B to 35% B over 60 min. The flow rate through the column was 250 nL per minute.

### Mass spectrometry

The instrument used for mass spectrometry was an LTQ Orbitrap Velos Pro (Thermo Fisher) equipped with a Thermo LTQ nanospray source, which was operated at an ion spray voltage of 1.8 kV and a heated capillary temperature of 275°C. Full scan mass data were obtained between 400–1,800 Da and the Orbitrap resolution was 30,000. The Orbitrap was operated in data dependent acquisition mode with dynamic exclusion (120 s). Twenty most intense ions above the minimum signal threshold with charge states greater than or equal to 2 were selected for low-energy CID in the ion trap. Other operating parameters included a minimum signal threshold of 25,000 and an activation time of 30.0 ms.

### Proteomics data analysis

Raw data files were processed using the Mass Matrix Conversion tool to generate Mascot generic files (MGFs) for the protein database search. Mascot 2.2 was used as a search engine and NCBI Mascot search results were imported into Scaffold.

### Western blot

Cheesy enamel matrix from 1 to 8 days postnatal mouse molars was scraped off and the proteins were homogenized and extracted using sodium dodecyl sulfate-polyacrylamide gel electrophoresis (SDS-PAGE) sample buffer. Equal amounts of the extracted proteins were loaded and separated on a 10% SDS-PAGE gel. From the gel, proteins were transferred to a polyvinylidene difluoride (PVDF) membrane in a semi-dry blotting apparatus at 18 V for 40 min. The membrane was blocked for 1 h with 5% milk powder after which it was incubated with anti-AMEL (1:200, custom made full-length), anti-AMBN (ab72776 1:200, Abcam), anti-ENAM (sc-33107, 1:100, Santa Cruz), anti-MMP20 (ab84737, 1:50, Abcam), and anti-carbonic anhydrase 2 (ab191343, 1:100, Abcam) primary antibodies for 1 h. Following primary antibody incubation, the membrane was washed three times with washing buffer (TBS-T) for 15 min each and then incubated with HRP conjugated secondary anti-chicken, anti-mouse, or anti-rabbit antibodies. To detect HRP, a chemiluminescent substrate (Thermo Scientific) was used. Positive bands were quantitatively assessed using densitometry analysis using the Image J software.

### Statistical analysis

For X-ray diffraction and ATR-FTIR studies, enamel matrix from four different mice of the same position and developmental stage was pooled, and pooled enamel matrix was used for further analysis. Mass-spectroscopy data and proteomics analysis were based on separately collected biological quadruplicates. All other data (thickness measurements and Western blot analyses) were based on triplicates. Data analysis was performed using SPSS software. Statistical significance was assessed using the non-parametric Mann-Whitney *U*-test, and the significance level was set at *p* < 0.05.

## Results

### Increasing thickness and birefringence of the developing enamel matrix

First mandibular molars of 1, 2, 3, 4, 5, 6, 7, and 8 day postnatal mice (Figures 1A–H) were dissected from alveolar bone crypts (Figure 2E) to characterize the developing enamel matrix. Stereo micrographs documented a continuous increase in enamel matrix thickness from 1–2 μm (1 day postnatal) to 75 μm (6–8 days postnatal) based on measurements of the enamel matrix thickness of the distal slope of the central major cusp, while the overall length of the tooth did not increase (Figures 1A–H, 2F). Analysis of 8 days postnatal molars between crossed polarizers revealed changes in matrix color pattern when analyzers were rotated in 90 degree intervals indicative of birefringence (Figures 2A–D). The soft and pliable consistency of the enamel matrix allowed for mechanical separation from the underlying dentin layer using a scalpel (Figures 2G,H).

### Single-crystal powder sample X-ray diffraction analysis of the postnatal enamel matrix yields calcium phosphate diffraction patterns on postnatal days 1 and 2, and apatite diffraction patterns from postnatal day 4 onward

**Figure 3:**
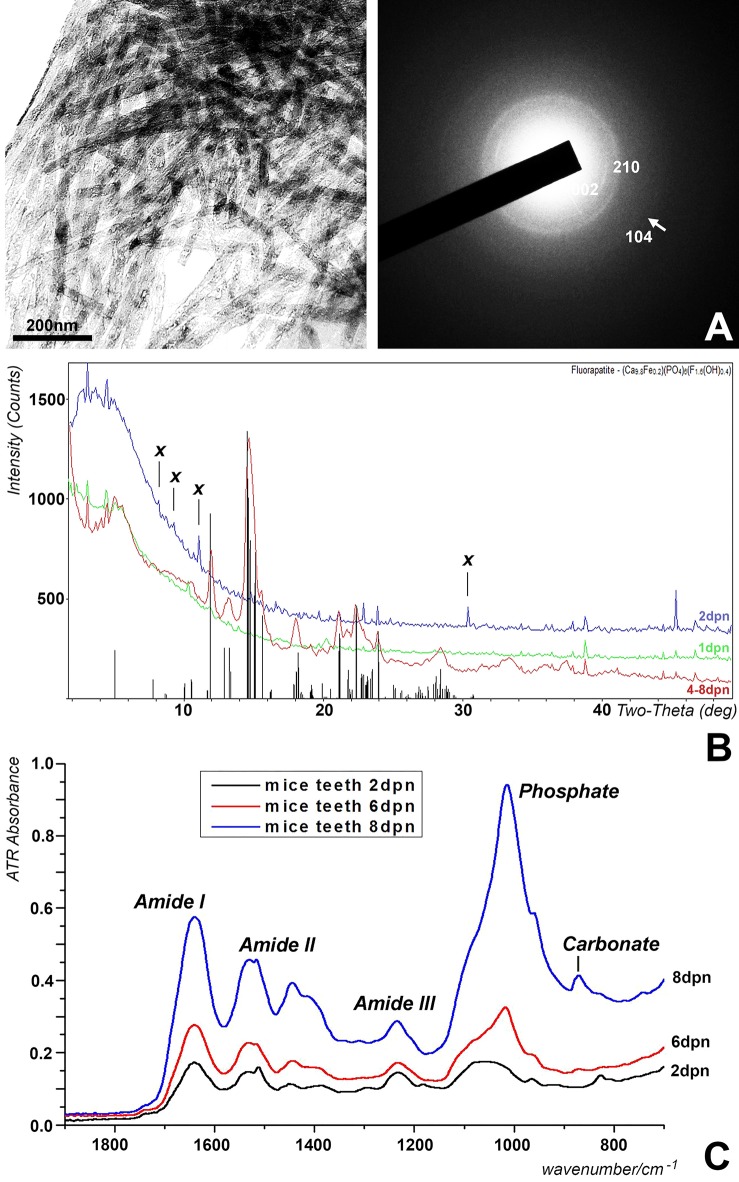
Analysis of the postnatal mouse molar enamel mineral layer. **(A)** Transmission electron microscopy of 4 days postnatal mouse molar enamel matrix revealed bundles of thick apatite crystals with diffraction rings in the 002 and 210 planes, and a faint diffraction ring in the 104 plane, indicative of hydroxyapatite. **(B)** X-ray powder diffraction analysis of enamel matrix preparations from the distal slopes of 1 day postnatal (1 dpn), 2days postnatal (2 dpn), 4, 6, and 8 days postnatal (4–8 dpn) mouse mandibular molars. The vertical bars at the base of the figure illustrate the partially fluoride substituted hydroxyapatite powder diffraction pattern with the height of the bars representing relative peak intensities. Unique unmatched peaks in the spectrum of 2 days postnatal samples were marked by an X. Only the 4–8 dpn samples matched hydroxyapatite powder diffraction standards. **(C)** Fourier-transform infrared spectra of developing enamel matrix preparations from the distal slopes of 2 days postnatal (2 dpn), 6 days postnatal (6 dpn), and 8 days postnatal (8 dpn) mouse mandibular molars. Peaks corresponding to vibrations for phosphate, carbonate, proteoglycan, and amide I–III are labeled individually.

Previous studies have indicated that the mineral phase of mouse molar enamel transitions from calcium carbonate, tri- and octacalcium phosphate precursors to partially fluoride substituted hydroxyapatite (Diekwisch et al., 1995; Diekwisch, 1998; Gopinathan et al., 2014). To determine at what stage the mineral phase of the entire postnatal mouse molar enamel matrix converts from calcium phosphate precursor stages to apatite (Figure 3A), dried enamel matrix preparations from developing mouse molars were subjected to single-crystal, powder sample X-ray diffraction analysis. Mineral phase analysis on days 1 and 2 revealed well-defined, weak-intensity peaks that only partially matched those of the apatite standard pattern and were indicative of a calcium phosphate precursor (Figure 3B). In contrast, samples from postnatal days 4–8 yielded partially fluoride substituted hydroxyapatite diffraction patterns based on powder diffraction standards (Hughes et al., 1991)(PDF# 73-9797) (Figure 3B). Four peaks labeled as X could not be matched to any ICDD data base pattern (Figure 3B).

### Attenuated total reflectance spectra (ATR) demonstrated enhanced secondary structure and increased phosphate and carbonate incorporation into the enamel matrix from postnatal day 2 to day 8

The ATR spectra were measured for teeth obtained at four stages of development (2, 4, 6, and 8 days postnatal, labeled as 2, 4, 6, and 8 dpn) of which 2, 6, and 8 dpn are shown in Figure 3C. The 4 dpn spectrum exhibited spectral characteristics similar to the 2 and 6 dpn spectra. The large band at ~1,650 cm^−1^ and the following one at ~1,540 cm^−1^ represent the amide I and II bands of enamel matrix proteins. The peak at ~1,237 cm^−1^ included the amide III band and likely other sources such as PO_2_ type modes due to its intensity. The strong peaks at 1,018 cm^−1^ with a shoulder at 1,105 and 958 cm^−1^ that emerged after 6 days was assigned to characteristic phosphate peaks (ν_3_ PO_4_ mode and ν_1_ PO_4_ stretching IR mode) (Antonakos et al., 2007; Leventouri et al., 2009). The peak at 872 cm^−1^ was representative of the ν_2_ CO_3_ band. Based on previous studies, we have assigned the weak shoulders at 1,520 and 880 cm^−1^ that appear after 6 days of development to A-type carbonate substitution (Elliot, 1994) and the increased peaks at 1,405 and 1,445 cm^−1^ to B-type carbonate substitution (ν_3_ CO_3_ mode) (Vignoles-Montrejaud, 1984). The major developmental changes in the spectrum when comparing 2, 4, 6, and 8 dpn enamel included the substantial elevation of the phosphate and carbonate bands (800–1,100 cm^−1^ and 1,400–1,480 cm^−1^) and the increased peak height of the amide I and II bands (1,650 and 1,540 cm^−1^, respectively), between postnatal day 6 and day 8.

### Proteomics demonstrated unique changes in the enamel organ/enamel matrix protein complex between postnatal days 2, 4, and 6

**Figure 4:**
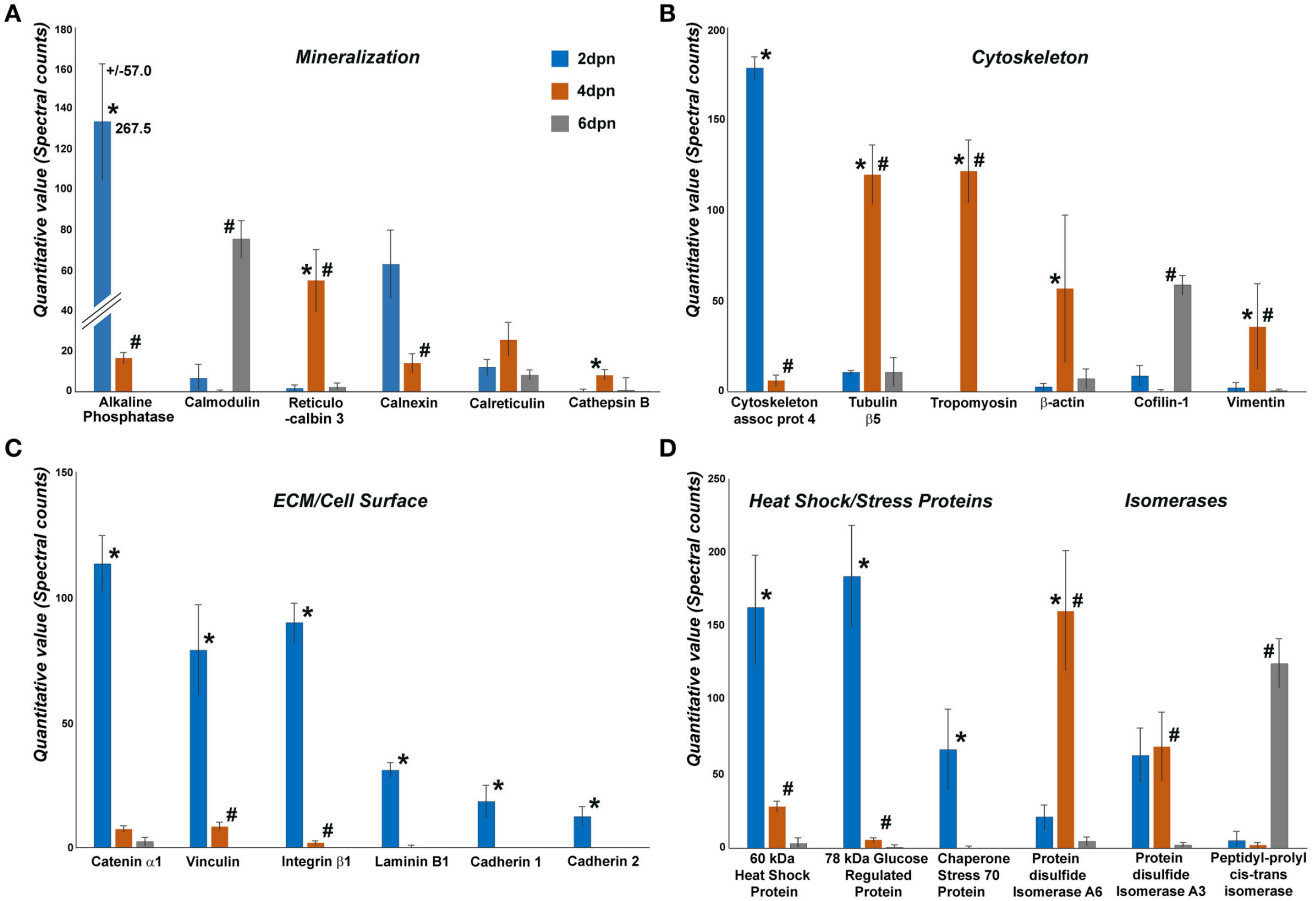
Comparison between spectral counts of individual proteins identified during postnatal mouse molar enamel development (days 2, 4, and 6 dpn)(99% probability, 2 peptide minimum). Proteins were grouped into four categories: **(A)** mineralization, **(B)** cytoskeleton, **(C)** extracellular matrix/cell surface, and **(D)** other. Six proteins with highest quantitative values in each group were chosen for display and further analysis. Orbitrap mass spectrometry is inherently biased to exclude hydrophobic proteins (Carroll et al., 2007; Griffin and Schnitzer, 2011), promoting a methodological focus on hydrophilic proteins at the expense of the hydrophobic proteins that occupy the majority of the developing enamel matrix. Spectral counts of 2 dpn (blue), 4 dpn (yellow), and 6 dpn (gray) enamel matrix were distinguished by color coding, and levels of significance were marked as ^*^p < 0.05, for comparisons between 2 and 4 dpn data, and ^#^*p* < 0.05, for comparisons between 4 and 6 dpn data.

Enamel organ proteomics analysis by nanoLC-MS/MS resulted in discrete peptide identification patterns distinguished between enamel organ/enamel matrix protein complex samples from postnatal days 2, 4, and 6. In these samples, individual proteins were identified using the Mascot search software and ranked based on quantitative Orbitrap counts. Mascot data analysis yielded five protein groups with high spectral counts (Figure 4), including (i) mineralization proteins, (ii) cytoskeletal proteins, (iii) extracellular matrix/cell surface proteins, (iv) stress proteins, and (v) isomerases. Within each group, individual proteins were ranked based on spectral count, and individual counts per day for each postnatal day (postnatal days 2, 4, and 6) were subjected to statistical analysis and displayed in Figure 4.

Among the biomineralization proteins, alkaline phosphatase 2 peaked on day 2 and significantly decreased by 0.9-fold (*p* < 0.05) from day 2 to day 4 while there was no alkaline phosphatase detected on day 6. Calmodulin was detected on day 2, decreased below the detection threshold on day 4, but once more rose to a significant peak on day 6. There was a 36-fold increase of reticulocalbin 3 levels from day 2 to day 4 (*p* < 0.05) but thereafter the reticulocalbin counts decreased significantly (*p* < 0.05) from day 4 to day 6. Calnexin levels decreased continuously from day 2 through day 6, including a significant decrease (*p* < 0.05) between days 4 and day 6. No expression for cathepsin B was observed at day 2, however the expression significantly peaked at day 4 (*p* < 0.05) and decreased again at day 6. In addition to the proteins displayed in Figure 4, peaks for the major enamel protein amelogenin continuously increased from day 2 to day 4 and day 6 (*p* < 0.05).

Four cytoskeleton related proteins displayed an increase in expression levels between days 2 and 4 and then decreased on day 6. Tubulin β5, β-actin and vimentin were detected at high levels on day 4 with a significant 10-fold (*p* < 0.05), 22-fold (*p* < 0.05), and 17-fold (*p* < 0.05) increase from day 2 to day 4, respectively, and a subsequent decrease between days 4 and 6 by 0.9-fold (*p* < 0.05). Cytoskeleton associated protein 4 on the other hand peaked on day 2, followed by a 0.9-fold (*p* < 0.05) decrease from day 2 to day 4, while cofilin-1 increased significantly from day 4 to day 6 (*p* < 0.05). Tropomyosin was not detected on days 2 and 6 but rose to significant levels on day 4 (*p* < 0.05).

All of the proteins related to extracellular matrix/cell surface displayed an identical trend of high levels on day 2, followed by a steep decrease through day 6. Catenin α1, vinculin, integrin β1, laminin B1, cadherin 1, and cadherin 2 all exhibited a significant decrease from day 2 to day 4 by 0.9-fold (*p* < 0.05), 0.9-fold (*p* < 0.05), 1-fold (*p* < 0.05), and 1-fold (*p* < 0.05), respectively. Vinculin and integrin β1 significantly decreased from day 4 to day 6 (*p* < 0.05). Laminin B1, cadherin 1, and cadherin 2 were only counted on day 2 and were not detected on days 4 and 6.

High levels of stress proteins 60 kDa heat shock protein and 78 kDa glucose regulated protein were detected on day 2, and then gradually decreased through day 6 with a 0.8-fold (*p* < 0.05) and 1-fold (*p* < 0.05) decrease, respectively, from day 2 to day 4 and a 1-fold (*p* < 0.05) decrease from day 4 to day 6 for both proteins. Chaperone stress 70 protein was present at high levels on day 2 but its expression level dropped significantly as it was not expressed either on day 4 (*p* < 0.05) or day 6. Protein disulfide isomerase A6 displayed a significant 7-fold (*p* < 0.05) increase from 2 to 4 dpn and a 1 fold decrease between days 4 and 6 (*p* < 0.05), while protein disulfide isomerase A3 peaked on day 4 and significantly decreased 1-fold (*p* < 0.05) on day 6. There was a remarkable 61-fold increase of peptidyl-propyl cis-trans isomerase between days 4 and 6 (*p* < 0.05).

### Western blot analysis of hydrophobic enamel proteins illustrated continuously increasing amelogenin levels from 1 dpn until 8 dpn, while enamelin peaked on days 1 and 2 dpn, and ameloblastin on days 1–5 dpn

**Figure 5:**
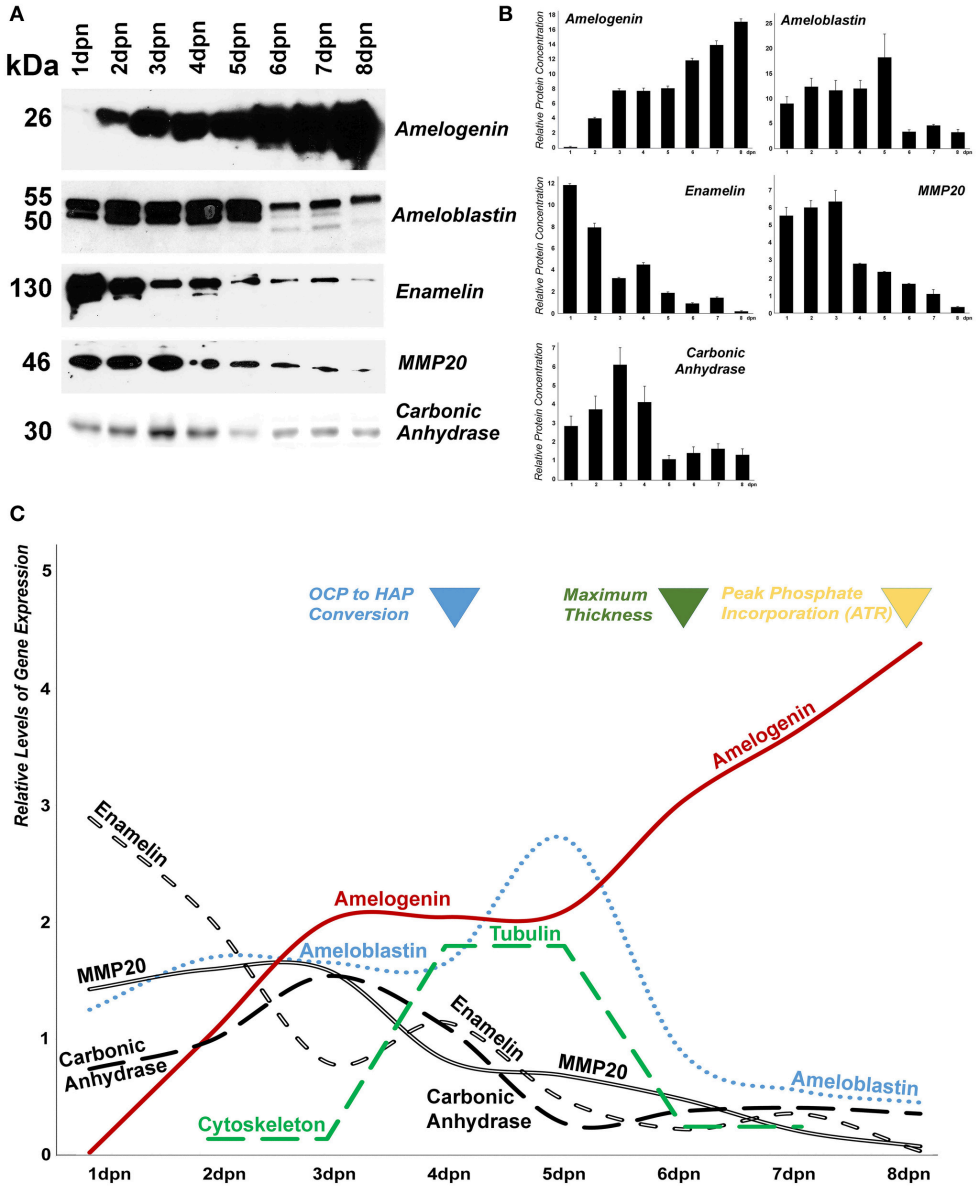
Changes in mouse molar enamel matrix protein levels from postnatal day 1–8. **(A)** Changes in amelogenin, ameloblastin, enamelin, MMP20, and carbonic anhydrase II, protein levels in enamel matrix preparations as revealed by Western blot. **(B)** Densitometric evaluation of protein levels in the Western blot shown in **(A)**. **(C)** Sketch correlating changes in protein levels with changes in the enamel mineral phase.

As an alternative strategy and because of the hydrophilic bias of our proteomics technology, known enamel organ/enamel matrix protein complex samples were assayed using classic Western blot methodology (Figures 5A,B). There was a continuous increase in the 26 kDa amelogenin band in our enamel organ extracts from postnatal days 1 to 8. Ameloblastin levels were high and unchanged between postnatal days 1 to 5, featuring both 50 and 55 kDa bands. Beginning with day 6, overall ameloblastin levels were reduced, only the upper 55 kDa band was present, and several bands of lesser molecular weight than the 50 kDa band were detected in addition to the 55 kDa band. Enamelin levels (130 kDa band) were relatively high on postnatal days 1 and 2, and gradually diminished thereafter. The 46 kDa band specific for the enamel protease MMP20 was present at relatively high levels from days 1 to 3 postnatal, and intensity decreased from 4 days postnatal onward. The pH regulator carbonic anhydrase II peaked between postnatal days 2 and 4, reaching a maximum on postnatal day 3 on our Western blot, while it was present at lesser quantities on postnatal days 1 and 5–8 (all Western blot data illustrated in Figures 5A,B).

## Discussion

Here we have used the developing mouse mandibular molar as a model system to track changes in protein and mineral composition during postnatal amelogenesis and to correlate findings from individual protein and mineral analyses to synthesize an integrated systems perspective of enamel formation in the mouse molar. The key benefit of the postnatal mouse molar model was the suitability of the distal slopes of first molar cusps to harvest sufficient quantities of fresh enamel matrix for proteomic and mineral composition analysis in daily increments. Moreover, the mouse mandibular molar has a long history as a model for morphogenesis, cytodifferentiation, and tissue specific biomineralization (Gaunt, 1955; Slavkin et al., 1976, 1988), allowing for cross-referencing between classic developmental biology data, mouse genetics models of amelogenesis, proteomics analysis databases, and the present systems biological study. The mouse molar model owes its popularity to its resemblance to the human molar in terms of completion of enamel formation following eruption and to the ubiquitous availability of mice and mouse genetic tools for experimental studies. In general, the mouse incisor would be equally attractive for studies of mammalian amelogenesis. However, for historical reasons and because of its larger size, the continuously erupting rat incisor has provided an alternative model for the study of enamel matrix and mineral composition during development (Schour and Massler, 1949; Robinson et al., 1979, 1981; Smith and Nanci, 1989). The rat incisor model relies on the preparation of sequential segments along the rat incisor labial surface representative of the entire sequence of amelogenesis from the youngest enamel at the root apex to the oldest enamel at the incisal edge (Robinson et al., 1997). We expect that amelogenesis in the continuously erupting rat incisor and in mouse molars of limited growth are highly similar because of the known similarities in the rat and mouse genomes, overall developmental patterns, and enamel mineral composition. In the present study, the mouse molar amelogenesis model was chosen because of the availability of mouse proteomics analysis tools and the feasibility of fresh enamel matrix preparation on successive days of postnatal amelogenesis.

We began our study by documenting the daily incremental increase in the thickness of enamel covering the distal slopes of the molar cusps between 1 dpn until 8 dpn. During this time, the semi-transparent enamel layer increased in thickness from 1 μm covering to 75 μm. Throughout those 8 days, the matrix was pliable and ideally suited for further biochemical analysis as it allowed for harvesting of the matrix and the attached enamel organ in bulk and in daily increments. The pliable nature of the developing enamel matrix has impressed naturalists and early biochemists since John Hunter's time (Hunter, 1778; Eastoe, 1979; Termine et al., 1980), and as a result has been termed cheesy or cheese-like by the early enamel biochemists (Alan Fincham, personal communication). The mouse molar enamel matrix retained its opaque, soft, and cheese-like consistency throughout postnatal development, with the exception of postnatal day 8, when the hardening of the matrix began. At that stage, matrix birefringence was at its peak, causing the enamel matrix to appear in alternating polarization colors depending on the angle between polarizing and analyzing filters. Earlier studies have focused on the anisotropy of the enamel matrix during the secretory stage (Keil, 1935; Schmidt, 1959; Spears et al., 1993; Do Espirito Santo et al., 2006). The highly polarized structure of the enamel matrix is evidence of the highly ordered enamel matrix structure as it provides a template for the highly ordered enamel mineral layer as a biomechanical buffer to occlusal loads and stresses.

X-ray diffraction analysis unambiguously identified enamel matrix diffraction patterns from day 4 to day 8 postnatal as partially fluoride substituted hydroxyapatite, and apatite electron diffraction patterns of 6 and 7 dpn mouse molar enamel were confirmed in the present study and in earlier electron diffraction studies (Diekwisch et al., 1995; Diekwisch, 1998). In contrast, X-ray diffraction patters from 1 to 2 dpn samples contained less prominent peaks that nevertheless matched the same overall partially fluoride substituted hydroxyapatite pattern of the samples from older enamel, and similarly, our earlier electron diffractions studies revealed fewer and less pronounced diffraction rings, suggesting that these earlier stages of enamel matrix maturation contain precursor phases of apatite maturation, such as octacalcium phosphate or tricalcium phosphate (Diekwisch et al., 1995; Aoba et al., 1998; Diekwisch, 1998). There were several non-identified peaks present in the 2 dpn sample, which were neither found in the 1 dpn sample nor in the 4–8 dpn samples, suggesting the presence of unusual intermediate phases during the transition from apatite precursors to fully mature hydroxyapatite. Together, these data demonstrate that the bulk of the distal slope first mandibular mouse molar enamel matrix converts from a lesser order of crystallinity into crystalline partially fluoride substituted hydroxyapatite between 2 and 4 dpn.

Our ATR data were interpreted according to previously published band assignments (Vignoles-Montrejaud, 1984; Elliot, 1994; Gadaleta et al., 1996; Boskey et al., 2006; Antonakos et al., 2007; Leventouri et al., 2009) and provide spectroscopic evidence for the structural conversion of the mouse molar enamel matrix from a mixed amorphous calcium phosphate/protein layer at 2 dpn to a crystalline hydroxyapatite structure featuring highly elongated crystals at 8 dpn. On a protein level, this change was accompanied by increased peak heights of the amide I and II bands, especially from postnatal day 6 to day 8, indicative of changes in protein secondary structure and increased rigidity of the peptide bonds between the carboxyl and the amino groups of two adjacent amino acids through an increase in the double bond character of the peptide bond, resulting in increased structural rigidity of the enamel protein matrix.

Our ATR data revealed dramatic changes in the phosphate region (800–1,100 cm^−1^) in the postnatal mouse molar develop enamel matrix. Specifically, our data demonstrated a transition from a contoured plateau (1,000–1,100 cm^−1^) indicative of amorphous calcium phosphate at 2 dpn to a single sharp and highly elevated peak (1,015 cm^−1^) with shoulders at 1,105 and 958 cm^−1^ at 8 dpn representative of high crystalline hydroxyapatite with crystals featuring long c-axis dimensions (Gadaleta et al., 1996). These findings are in congruence with our previous studies suggestive of a gradual transition from amorphous calcium phosphate to highly ordered and elongated apatite crystals during amelogenesis through a process called Ostwald ripening (Diekwisch et al., 1995; Aoba et al., 1998; Diekwisch, 1998).

Moreover, there was strong evidence for carbonate substitution in the enamel matrix between postnatal days 6 and 8, as the peaks at 880 cm^−1^ (A-type carbonate) and 1,405 and 1,445 cm^−1^ (B-type carbonate) indicated. Carbonate is known to replace phosphate in biological apatites (Zapanta-Legeros, 1965; Wopenka and Pasteris, 2005), affects its physical properties, including decreased crystallinity and increased c-axis length (Fleet et al., 2004; Wopenka and Pasteris, 2005; Boskey et al., 2006) and mechanical properties, such as decreased hardness and Young's modulus (Morgan et al., 1997; Xu et al., 2012).

Our proteomic analysis provided only low counts for classic enamel proteins, which are known to be of hydrophobic nature (Eastoe, 1965), even though its most prominent member, amelogenin, comprises 80–90% of the developing enamel matrix protein composition (Fincham et al., 1999). This result is characteristic for unmodified proteomic studies, which have a systematic bias against hydrophobic proteins and membrane proteins and favor hydrophilic components instead (Santoni et al., 2000; Chandramouli and Qian, 2009; Josic, 2014). To address the systematic scarcity of hydrophobic proteins in proteomic datasets, classic Western blot studies of enamel protein expression from days 1 to 8 postnatal were performed. These studies demonstrated a continuous increase in amelogenin from day 1 to 8, relatively high levels of ameloblastin from days 1 to 5, peak enamelin protein level peaks on day 1 and 2, relatively high levels of MMP20 from days 1 to 3, and relatively high levels of carbonic anhydrase between postnatal days 2 and 4. These data underscore the continuous presence of amelogenin during the secretory stages of amelogenesis (Termine et al., 1980), while ameloblastin, enamelin, and MMP20 are only prominent during early secretory stage enamel development, possibly establishing a patterning template for enamel crystal and prism growth (Bartlett, 2013; Pugach et al., 2013; Zhu et al., 2014; Prajapati et al., 2016). Our Western blot data provided evidence for relatively high levels of carbonic anhydrase during early enamel development (days 1–4), indicative of a role for pH adjustment during initial crystal nucleation, while other regulators may be involved in the pH regulation during the massive elevation of phosphate and carbonate during late-stage crystal growth. In addition, the continuous increase in amelogenin during postnatal enamel development was also verified by our proteomics study, albeit at a lower level of counts due to its hydrophobicity. Three of the proteins analyzed in Figure 4 (Calmodulin, Cofilin-1, and Peptidyl-prolyl cis-trans isomerase) featured higher levels of expression on days 2 and 6, and a substantial decline in protein counts on day 4. We interpret these data to indicate that such proteins may have dual functions during enamel ion transport and enamel crystal growth.

Disregarding the moderate counts for classic enamel proteins such as amelogenin, ameloblastin, and enamelin, our proteomics analysis detected a number of other proteins relevant for enamel mineralization during our 2–6 days postnatal mouse molar enamel matrix sampling window. Among these was the protein with the highest number of counts in our analysis, alkaline phosphatase, which peaked at day 2 during the early secretory stage. At the onset of amelogenesis, alkaline phosphatase may be involved in transporting phosphate from blood vessels near the stratum intermedium into the enamel organ by increasing local phosphate concentrations in the stratum intermedium via hydrolysis of phosphorylated substrates. Phosphatase mediated hydrolysis of pyrophosphate may also be involved in the generation of other phosphorylated macromolecules (Woltgens et al., 1995; Liu et al., 2016). The other mineralization-related protein with an early peak at 2 days postnatal was the protein folding chaperone calnexin, which may play a role in the prevention of endoplasmic reticulum stress due to misfolded enamel proteins or in calcium transport (Wang et al., 2005; Brookes et al., 2014). Calreticulin, which peaked at postnatal day 4, is another enamel protein with a dual function misfolded protein processing and calcium transport (Somogyi et al., 2003) that was detected in our proteomics analysis. Two other calcium transport proteins, calmodulin and reticulocalbin-3, peaked on day 6 (maturation stage, calmodulin) or on day 4 (late secretory stage, reticulocalbin-3). Calmodulin and reticulocalbin are capable of binding calcium using an EF-hand motif, and this function may be related to the protein-mediated transport of calcium ions through the ameloblast cell layer. The most prominent enzyme in our proteomics analysis of mouse molar enamel organs was the lysosomal cysteine protease cathepsin B, which peaked on day 4 postnatal. Cathepsin B is an important lysosomal enzyme of the enamel matrix (Al Kawas et al., 1996; Tye et al., 2009) that has been shown to enhance the activity of other proteases such as metalloproteinases and cathepsin D (Hammarström et al., 1971; Blair et al., 1989). As such, cathepsin B may promote the proteolytic degradation of the enamel matrix by enhancing the activity of MMP20 and cathepsin D.

Four of the six high-scoring cytoskeletal proteins in the enamel organ, including actin, tubulin, tropomyosin, and vimentin isoforms, peaked at postnatal day 4 during the late secretory stage. Increased presence of cytoskeletal proteins during the late secretory stage is likely indicative of their role in cell polarization and vesicular secretion (Manneville et al., 2003; Neco et al., 2003; De Lisle, 2015). Highly specific actin and tubulin immunoreactivity in secretory ameloblasts has been reported earlier (Diekwisch, 1988; Kero et al., 2014). Cytoskeleton associated protein 4 (CLIMP63) is actually a transmembrane protein that is instrumental in anchoring the endoplasmic reticulum to microtubules (Vedrenne et al., 2005), and its early secretory stage expression matches that of other transmembrane proteins reported here (cadherin, catenin). In contrast, Cofilin-1 levels only became elevated during the resorptive maturation stage (6 dpn), which may be explained by its role in actin depolymerization (Maciver and Hussey, 2002; Morita et al., 2016). Vimentin has long been hailed as an intermediate filament protein marker of mesenchymal tissues (Kidd et al., 2014). However, our present study reports strong evidence for vimentin signals in the enamel organ, lending support to earlier studies about transitory vimentin expression in the stellate reticulum (Kasper et al., 1989; Kero et al., 2014). Together, our proteomic data provide strong evidence for high levels of cytoskeletal proteins at the late secretory stage, likely related to their role in ameloblast polarization and enamel matrix secretion.

Our proteomics data indicated that six high-scoring extracellular matrix/cell surface molecules identified in the present study all peaked on day 2 postnatal at the onset of the secretory stage. This group included the classic extracellular matrix molecule laminin, the integrin β1 cell surface receptor, the β-integrin binding extracellular matrix adhesion molecule vinculin, and three cell adhesion molecules of the catenin/cadherin complex. The presence of laminin as part of the ameloblast basal lamina at the dentin-enamel junction has been demonstrated to play a role in terminal odontoblast differentiation (Lesot et al., 1981), and this basal lamina is removed during further ameloblast differentiation, presumably facilitating enamel deposition in sarcopterygian vertebrates (Diekwisch et al., 2002). Based on previous studies, we speculate that both the adherens junction protein vinculin and its binding partner β-integrin function to establish the sliding interface between secretory ameloblasts that allows for ameloblast cell movements during prism formation (Kubler et al., 1988; Nishikawa et al., 1988, 1990; Xu et al., 1998; Saito et al., 2015). Three of the six significantly upregulated proteins belonged to the cadherin-based adherens junction complex, namely catenin α1 and cadherins 1 and 2. Catenins are known to interact with cadherins (Rangarajan and Izard, 2013) and provide a link between adherens junctions and the actin cytoskeleton (McCrea and Gu, 2010). In ameloblasts, E-cadherin appears to be involved in ameloblast polarization (Terling et al., 1998) and β-catenin is essential for ameloblast movement (Guan et al., 2016). A switch between E-cadherin and N-cadherin has been documented in ameloblasts that slide by each other to form decussating enamel rod patterns (Guan et al., 2014). Together, the high levels of adherens junction and matrix proteins in the early enamel organ are indicative of the involvements of these proteins at the early secretory stage of amelogenins.

Three heat shock/stress proteins also were among the high-scoring proteins that peaked at the onset of the secretory stage. These proteins included the 60 kDa heat shock protein (Hspd1), the 78 kDa glucose regulated protein (Grp-78), and the chaperone stress 70 protein (Hsp70). All three of these proteins are involved in macromolecular assembly, the prevention of misfolding, as well as the prevention of aggregation. One of the major challenges of amelogenesis is the transport of amelogenin, a protein prone to self-assembly (Zhang et al., 2011), through the ameloblast cells. It is likely that both Grp-78 and Hsp70 may be involved in the prevention of amelogenin self-assembly inside of the ameloblast cell body, as both proteins are known to prevent protein aggregation (Wegele et al., 2004; Mayer and Bukau, 2005), while Hspd1 might rather function to prevent amelogenin misfolding (Xu et al., 2006).

Three isomerases joined the list of high-scoring enamel proteins, including the protein disulfide isomerases A3 and A6, and the peptidyl-prolyl cis-trans isomerase. Disulfide isomerases (PDIs) catalyze protein folding by facilitating disulfide bond formation and arrangement (Kersteen and Raines, 2003), a process that apparently takes place during the entire secretory stage, as PDI peaks in our proteomics data indicate. In contrast, peptidyl-prolyl cis-trans isomerase interconverts the trans-isomers of newly synthesized peptide bonds into cis-isomers of higher biological activity (Herzberg and Moult, 1991; Pal and Chakrabarti, 1999; Balbach and Schmid, 2000; Shaw, 2002). Peptidyl-prolyl cis-trans isomerase function may play a role in the conformational modification of amelogenin and enable its role as a molecular hinge in the promotion of crystal growth (Delak et al., 2009).

In conclusion, this integrative proteomic/cell biological analysis of postnatal mouse molar enamel development identifies many of the substantial changes in the enamel matrix protein and mineral phase that take place during postnatal mouse molar amelogenesis, including (i) relatively high levels of matrix protein secretion during the early secretory stage on postnatal day 2, (ii) conversion of calcium phosphates to apatite, peak protein folding and stress protein counts, and increased cytoskeletal protein levels such as actin and tubulin on day 4, as well as (iii) secondary structure changes, isomerase activity, highest amelogenin levels, and peak phosphate/carbonate incorporation between postnatal days 6 and 8 (Figure 5C).

## Ethics statement

All animal experiments were approved by the IACUC committees at the University of Illinois, Chicago and Texas A&M College of Dentistry.

## Author contributions

MP, WZ, and TD wrote the manuscript. SG, TK, XL and TD designed the experiments. MP, HL, SD, LL,WZ, SP, and RD performed the experiments. MA designed and performed the statistical analysis.

### Conflict of interest statement

The authors declare that the research was conducted in the absence of any commercial or financial relationships that could be construed as a potential conflict of interest.
